# Resveratrol Effect on Adipose-Derived Stem Cells Differentiation to Chondrocyte in Three-Dimensional Culture

**DOI:** 10.15171/apb.2020.011

**Published:** 2019-12-11

**Authors:** Ghazal Keshavarz, Cyrus Jalili, Mona Pazhouhi, Mozafar Khazaei

**Affiliations:** ^1^Student Research Committee, Kermanshah University of Medical Sciences, Kermanshah, Iran.; ^2^Fertility and Infertility Research Center, Health Technology Institute, Kermanshah University of Medical Sciences, Kermanshah, Iran.

**Keywords:** Adipose stem cells, Chondrocyte, Resveratrol, Three-dimensional culture

## Abstract

***Purpose:*** Adipose stem cells (ASCs) are pluripotent cells with the ability of self-renewal and differentiation into different types of mesenchymal cells. As cartilage repair is difficult due to lack of blood capillary, resveratrol (Res) is a polyphenolic compound with diverse biological properties to be possibly used in this case. The aim of the present study was to investigate the effect of Res on differentiation of ASCs into chondrocyte in a three-dimensional (3D) culture model.

***Methods:*** Subcutaneous adipose tissues were prepared and digested enzymatically, and passed through cell strainer. ASCs were harvested in the fourth passage, and divided into five groups. The control group received chondrogenic differentiation medium (CDM) while the experimental groups received CDM plus different doses of Res (1, 10, 20, and 50 µM) for 21 days. Expression of cartilage specific genes and Sirtuin1 (SIRT 1), cell viability, apoptosis and ferric reducing antioxidant power (FRAP) were detected using reverse transcription polymerase chain reaction (RT-PCR), MTT assay, TUNEL and acridine orange/ethidium bromide (AO/EB) staining. One-way ANOVA and non-parametric Mann-Whitney U test were used for data analyses.

***Results:*** ASCs were differentiated to chondrocyte by CDM in a three-dimensional culture. 10 and 20 µM doses of Res showed the most proliferating effect on ADSCs. The SIRT 1 genes expression and FRAP level also increased significantly compared to the control group (*P*<0.05). Also, OD of cell increased whereas apoptosis decreased.

***Conclusion:*** 3D culture was a suitable condition for ASCs differentiation to chondrocyte, and lower doses of Res exert proliferation effect on ASCs.

## Introduction

Stem cells are undifferentiated cells with long-term self-renewal and differentiation ability into many types of cells derived from three embryonic germ layers.^1^ Embryonic stem cells are the pluripotent cells with ethical limitations in application. For this reason, in the laboratory research, adult stem cells are used. In the early 1970s, Friedenstein isolated the mesenchymal stem cells (MSCs) from the bone marrow.^2^ Adult stem cells are a population of multipotent cells found in different tissues such as bone marrow, skeletal muscle, brain, umbilical cord blood, peripheral blood, skin and adipose tissue. They have surface markers of CD90, CD105, CD73, CD106, CD29 and CD44 and lack of the hematopoietic markers such as CD34 and HLA-Dr.^3^

One of the potential of tissue repair is the presence of stem cells which have high division power and differentiation capacity. They help to maintain tissue lining cells and survival of the adult blood system. Adipose tissue consists of different cells, with adipocytes accounting for one third of them.^4^ This tissue is a main source of stem cells and one gram of it has about 5000 stem cells, which is 35 times more than the stem cells of bone marrow.^5,6^ Adipose stem cells (ASCs) are more common around blood vessels and are closely related to endothelial cells and pericytes.^7^

Stem cell isolation from adipose tissue is preferable to bone marrow due to higher cell numbers, easy access, and less invasion. For this reason, it has been considered in recent decades and is a very good alternative to damaged pancreatic islets cells,^8^ tendons, ligaments and injured articular cartilage.^9^ Also, transplantation of adipose tissue to various organs reduces fibrosis, increases the number of vessels, and modifies the collagen structure in transplanted tissues.^10^

Articular cartilage has unique biological and biomechanical properties. The reduction and destruction of this tissue in the weight bearing joints causes pain, stiffness, swelling, locking of the joints and severe motor limitation for the patient together with high cost of treatment. Due to this fact that cartilage has no blood or lymphatic vessels and nerves, chondrocytes have low division activity and little capacity for their reconstruction and repair,^11^ leading to tissue fibrosis and providing conditions for the development of osteoarthritis.

In recent years, tissue engineering has been widely used to repair cartilage, and its main components are scaffolds and various cell types such as ASCs. The selected scaffolds should be compatible with the environment, free from toxicity and inflammatory cells stimulation, resistant to physiological pH and body temperature in order to allow the cells to adhere, grow, and differentiate.^12^ In addition to the types of scaffold, various compounds can contribute to the differentiation and growth of the chondrocyte.

Resveratrol (Res) (3, 5, 4’-trihydroxystilbene) is one of the polyphenolic and phytoalexin compounds which was first identified in 1940 in the root of the white hellebore (*Veratrum grandiflorum*). It is also abundant in many other herbs such as red grapes, peanuts, eucalyptus, blueberries, melons and black raspberries.^13,14^ Res is synthesized by plants in response to stress and infection. This compound attaches to estrogen receptors and activates transcription of other genes.^15^ It also has many other properties such as anti-tumor and anti-inflammatory effects, anti-aging and neuroprotective qualities,^16^ chemotherapy properties^17^ and endometriosis suppression features.^18^

Sirtuins are a member of class III histone deacetylases (HDACs) which is dependent on NAD^+^, and seven types of them have been reported in humans.^19^ Sirt1 has been known as an anti-aging factor and plays an important role in preventing chondrocytes apoptosis and osteoarthritis pathogenesis.^20^ It has different functions such as regulation of lipid metabolism and adipogenesis.^21^ Sirt1 interferes with damage-related DNA responses and is associated with increasing age and carcinogenesis. This protein is the inhibitor of diseases such as colon cancer^22^ and sarcoma, lymphoma, teratoma, carcinoma^23^ and age-related spontaneous tumors.^24^ It is believed that there is a direct relationship between Res and Sirt1, meaning that Res existing in diet increases the survival of cells by inducing the *Sirt1* gene expression.^25,26^

The aim of the present study was to investigate the effect of Res on differentiation of ASCs into chondrocyte in 3D culture and to evaluate cell survival, apoptosis, total antioxidants capacity and *Sirt1* gene expression.

## Materials and Methods

In this *in vitro* experimental study, subcutaneous adipose tissues were taken from patients (20-40 years) during liposuction in a sterile phosphate-buffered saline (PBS) solution. The adipose tissues were cut into small pieces and after washing with PBS, they were chopped with sterile blade and incubated in collagenase type I (2 mg/mL) solution for 60 to 90 minutes. After enzymatic digestion, the cell suspension was passed through 70 and 40 µm filter mesh (cell strainer) to eliminate undigested tissue fragments.

The suspension was centrifuged at 2000 rpm for 10 minutes. The cell pellet (stromal vascular fraction) was re-suspended in fresh DMEM (Dulbecco’s Modified Eagle Medium) containing 10% fetal bovine serum and 1% antibiotic, and was subsequently transferred to a culture flask. The culture flask was maintained in a 5% CO2 incubator at 37°C. After removing the floating cells in the first 24 hours, the medium was changed every two days, and once reaching a density of 70%-80%, the cells were passaged until the 4^th^ passage.

### Flow cytometry technique

Flow cytometry is one of stemness confirmation methods for determining the mesenchymal markers such as CD105, CD73 and non-mesenchymal marker such as CD45. Isolated ASCs (passage 4) were washed with flow cytometry (FCM) buffer [PBS containing 0.5% bovine serumalbumin (BSA)] two times. The purity of MSCs was determined using anti-CD105-FITC, anti-CD73-PE and anti-CD45-FITC. Then, MSCs were incubated with 10 μL of each antibody or isotype antibody for 45 min at 4^◦^C. The cells were subsequently washed three times with FCM buffer, fixed with 1% paraformaldehyde and subjected to FCM (FACS Calibur, Beckman Dickinson, San Jose, CA). Data of FCM were analyzed by FCS Express V3 Software (De Novo Software, Los Angeles, CA).

### Three-dimensional culture

Fibrin scaffold was used to carry out the three-dimensional (3D) culture. In this method, fibrinogen (3 mg/mL) was dissolved in a M199 medium containing ASCs and was addedto wells of 24 wells plate (0.5 mL/well). Then, 15 µL of thrombin (Stago) was added to each well, and the culture dish was placed in the incubator. After formation of fibrin jelly, media of different groups were added for 21 days and changed every three days.^27^

### ASCs differentiation into chondrocyte

ASCs were incubated with chondrogenic differentiation medium (CDM) consisting of high-glucose DMEM, insulin-transferrin-selenium 1%, dexamethasone (100 nM), ascorbic acid 2 phosphate (50 μg/mL), BSA (1.25 mg/mL) and TGF-β3 (10 ng/mL).The cells were divided into five groups. The control group was treated only with CDM, but the experimental groups (2nd to 5th) were treated with CDM containing one of 1, 10, 20 and 50 µM doses of Res (Sigma) for 21 days. The effects of different doses of Res on morphology, growth and differentiation of the ASCs in a 3D culture were investigated and compared with the control group.

### Alcian blue staining

Alcian blue staining was used for chondrocyte confirmation. In this staining, the cells were fixed with 4% paraformaldehyde and stained with alcian blue (1 g/100 mL of 0.1 M hydrochloric acid with pH = 1) and incubated for half an hour.The wells were rinsed three times with 0.1 M HCl and finally washed with distilled water. The blue color indicates the produced proteoglycan compounds by chondrocytes.

### MTT assay

MTT [3- (4, 5-dimethylthiazol-2-yl) -2, 5-difenyltetrazolium bromide] assay was used for measuring the viability of cells. ASCs were cultured in 96-well plates (15 × 10^3^ cells per well), and after 72 hours of cells treatment with different concentrations of Res [0 (control), 1, 10, 20 and 50 μM], the medium was removed. Then 30 μL of MTT solution (Roche, GmbH, Germany) (5 mg MTT dissolved in 1 mL PBS) was added to each well, and the cells were incubated for 3 hours in a dark condition. Then, 100 μL of dimethyl sulfoxide was added to dissolve the formazan crystals at room temperature on a shaker. The optical density (OD) at 570 and 630 nm wavelength was read using the ELISA reader.^28^

### TUNEL staining

Cells apoptosis was detected using a TUNEL Kit (Roche Diagnostics, Germany) by labeling the end of the 3́OH of the DNA fragments. The cells were incubated in a 96-wells plate for 72 hours with different doses of Res. Then, the medium was removed, and the cells were fixed with 4% paraformaldehyde solution for half an hour at room temperature. The cells were rinsed with PBS, and a solution of 0.1% Triton X-100 in 0.1% sodium citrate was used for 5 minutes on ice (4°C). Again, the cells were rinsed with PBS and 50 µL of TUNEL mixture containing the enzyme solution, and its label was added to each well at room temperature for an hour. The cells were rinsed with PBS, and the propidium iodide (PI) solution was used for specific staining of the cells for 20 minutes. Healthy cells were identified as opaque red cells from apoptotic cells in gloss red. The work was done in the dark, and after three times of rinsing cells with PBS, they were observed through a fluorescence microscope.^29^ The apoptotic index of the cells was calculated using the following formula

Apoptotic index = number of apoptotic cells/total number of cells ×100

### Acridine orange/ethidium bromide (AO/EB) staining

This method was used to distinguish dead cells (morphological changes) from live ones. An amount of 100 μL of AO/EB mixture (100 μg/mL of each dissolved in PBS) was used for 20 minutes. After washing with PBS, the cells were observed through a fluorescence microscope (Nikon, Germany). In this staining, the AO passes through the plasma membrane of all cells and emits a green fluorescent light, but EB only passes through the membrane of damaged cells and emits a red fluorescent light. Due to the predominance of EB in relation to AO, the nuclei of live cells are integrated and green while the nuclei of primary apoptotic cells have a split chromatin and are yellow but the nucleus of cells with more advanced apoptosis show orange and fragmented chromatin.^30^

### Ferric reducing antioxidant power (FRAP)

Total antioxidant capacity was measured using FRAP technique. First, the FRAP working solution containing 25 mL of acetate buffer (300 mM with pH 3.6) was mixed with 5 mL of the tripyridyl, 1, 3, 5 triazine substance in chloride acid (40 mM). Then, 5 mL of iron chloride (20 mM) was added to the solution. 1.5 mL of the prepared solution was added to 200 μL of the samples and placed in a hot bath for 15 minutes at 37°C. The absorption changes were measured at 593 nm by a spectrophotometer. The FRAP value was converted to μM unit using the FeSo4.7H2o standard.^31^

### Semi-quantitative PCR (Reverse Transcription-PCR)

To analyze *Sox2, Oct4, Nanog, Sox9,* collagen II (*COL2A1*), aggrecan (*ACAN*) and *Sirt1* genes expression, a semi-quantitative polymerase chain reaction (PCR) was performed. 1 μL of cDNA was used as templet in RT-PCR. It was added to 12.5 μL of 2x Master Mix RED (1.5 mM MgCl2) (Amplicon Inc.), including 150 mM Tris-HCl pH 8.5, 40 mM (NH4)2S04, 3 mM MgCl2, 0.2% Tween^®^ 20, 0.4 mM of each dNTP, 0.2 units/µL Amplicon Taq DNA polymerase, inert red dye and stabilizer), 1 µL of each primer (10 μM), and up to 25 μL nuclease free water. Amplification was carried out using a thermal cycler (Eppendorf AG 22331, Hamburg, Germany), and conditions of RT-PCR amplification were as follows: an initial denaturation at 94°C for 5 minutes, 30 cycles of three-step PCR consisting of 94°C for 20 seconds, 60°C for 25 seconds and 72 °C for 45 seconds and final extension at 72°C for 10 minutes. The RT-PCR product was applied for electrophoresis agarose gel along with molecular weight markers.

### Real time PCR

To extract total RNA from the cells, the fibrin gel was harvested, and the cells were released by chopping and centrifuged at 2500 rpm for 10 minutes. The supernatant was discarded, and the next steps were carried out in accordance to the instructions in the GeneMATRIX Universal RNA Purification (EurX) kit. In the last step, to measure the amount of extracted RNA, NanoDrop 2000c Spectrophotometer (Thermo Fisher Scientific, Waltham, MA, USA) was used at a wavelength of 260/280.

For the synthesis of cDNA, 1 μg of extracted RNAs was used by the PrimeScript^TM^ RT Reagent Kit Reverse Transcription (TaKaRa, Dalian, China), and the steps were taken according to the kit’s instructions. Real time-PCR was performed with the ABI device (Step one) to evaluate whether Res altered the expression of the desired genes. Each reaction mixture contained 1 μL of cDNA, 1 μL of forward and revers primer (5pM) and 10 μL of Cyber Green’s mixer, which were taken together with distilled water at a volume of 20 μL .The specificity of the products was evaluated by comparing the melting curve, and the relative expression level of each gene was calculated by the 2^-ΔΔCT^ formula. The ACTB was used as a reference gene to measure the relative expression of the desired genes and was repeated three times for each group.Table 1 shows the specific primers that used in this study.

**Table 1:** Genes primer sequences

**Gene**	**Accession Number**	**Annealing temperature, °C**	**Size (bp)**	**Primer sequence**
*ACTB*	NM_001101	60	146	F: 5ˊ-TGACCCAGATCATGTTTGAGACC-3ˊ R: 5ˊ-CTCGTAGATGGGCACAGTGTGGG-3ˊ
*SOX2*	NM_003106	60	87	F: 5ˊ-CTCGCCCACCTACAGCAT-3ˊ R: 5ˊ-GCCTCGGACTTGACCACC-3ˊ
*POU5F1 (OCT4)*	NM_001173531	60	110	F: 5ˊ-AGTGAGAGGCAACCTGGAGA-3ˊ R: 5ˊ-ACACTCGGACCACATCCTTC-3ˊ
*NANOG*	NM_024865	60	158	F: 5ˊ-CAAAGGCAAACAACCCACTT-3ˊ R: 5ˊ-TCTGCTGGAGGCTGAGGTAT-3ˊ
*Sox9*	NM_000346	63	256	F: 5ˊ-TACGACTACACCGACCACCA -3ˊ R: 5ˊ-TTAGGATCATCTCGGCCATC -3ˊ
*COL2A1*	NM_001844	61.5	79	F: 5ˊ-GGCAATAGCAGGTTCACGTACA -3ˊ R: 5ˊ- CGATAACAGTCTTGCCCCACTT-3ˊ
*ACAN*	NM_001135	56	185	F: 5ˊ- CCAGGAGGTATGTGAGGA -3ˊ R: 5ˊ- CGATCCACTGGTAGTCTTG -3ˊ
*Sirt1*	NM-00114249801	60	110	F: 5ˊ-TCAGTGTCATGGTTCCTTGC-3ˊ R: 5ˊ-GTTCATCAGCTGGGCACCTA-3ˊ

### Statistical analysis

The statistical analysis of this study was done by SPSS software version 16 (SPSS Inc., Chicago, IL, USA) with mean ± SD. One-way ANOVA and non-parametric Mann-Whitney U test were used for data analysis. *P*< 0.05 was considered statistically significant.

## Results

**Figure 1:**
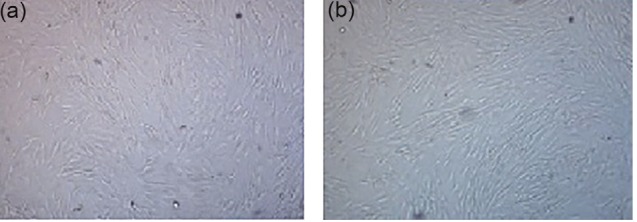
ASCs in second passage (a) and fourth passage (b): ×10.

After 24 hours of primary culture, the ASCs attached to culture flask with small, spheroid and transparent appearance. After one week, the cells became larger; their heterogeneity was decreased, and they acquired a fibroblastic-like form. When the cells reached 70%-80% confluency, they were passaged, and in the passages 3 and 4, they were uniformly homogeneous with spindle morphology (Figure 1).

### Purity of ASCs

**Figure 2:**
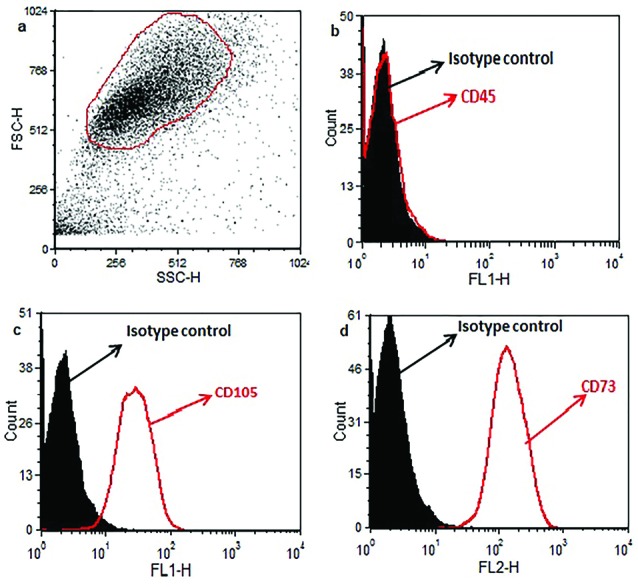
Data of flow cytometry analysis indicated that ASCs are positive for mesenchymal markers (CD105 and CD73) but not for CD45 (hematopoietic marker). The black-filled histogram shows stained ASCs with isotype control antibody. The red open histograms (Figure 2a) display CD45, CD105, or CD73 markers.

To determine the purity of ASCs based on the FCM technique, the cells were stained with mesenchymal and hematopoietic markers. According to Figure 2, ASCs did not express the hematopoietic marker of CD45 (Figure 2b), but they were positive for mesenchymal markers of CD105 and CD73 (Figure 2c, d). These results show that our isolated ASCs were pure from any hematopoietic cells.

### ASCs differentiation into chondrocytes

**Figure 3:**
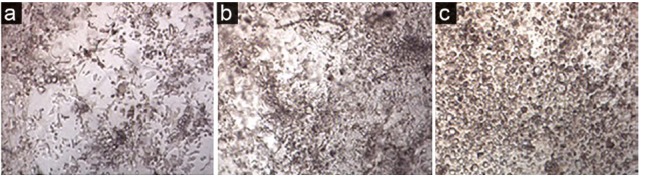
ASCs incubated in the CDM on days 7 (a), 14 (b) and 21 (c). ×4.

In the early days after ASCs incubation in the CDM, the cells were small with uniform morphology (Figure 3a), but over time, they became larger with round morphology (Figure 3b). During the third week of differentiation, all cells had a round or oval shape with the distinct cellular components (Figure 3c).

**Figure 4:**
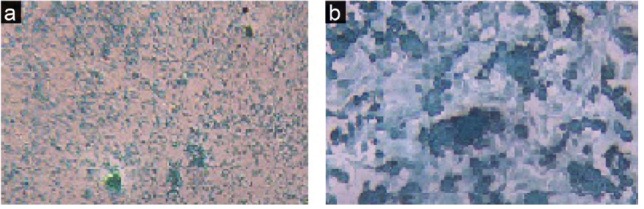
Alcian blue staining for chondrocytes, a: ×4 and b: ×10.

The cell morphology of the experimental groups that received CDM with different doses of Res (1, 10, 20, 50 μM) was similar to the control group, but their densities were different. 1 μM dose of Res did not have considerable effect on cells density, but the 10 and 20 μM doses increased the ASCs density; the cells were very clear spherically formed with distinct nuclei. The cells densities at 50 μM dose were lower than the control group, and they lost their spherical shape. Alcian blue staining (specific for chondrocyte cells) appeared blue due to proteoglycans secretion (Figure 4).

### MTT assay

**Figure 5:**
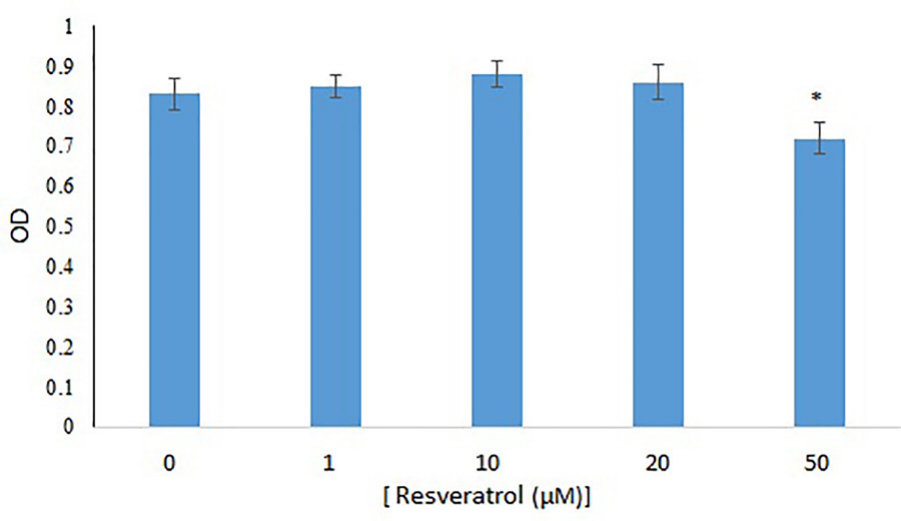
Cells OD rate were investigated using MTT assay with doses of 0 (control), 1, 10, 20 and 50 μM of Res after 72 hours of incubation compared with control group. The data were expressed as mean ± SD. Results were compared with the control group, *P*<0.05.

The results showed that the OD of the cells in the control groups and Res doses of 1, 10, 20 and 50 μM were 0.83 ± 0.0373, 0.85 ± 0.0287, 0.88 ± 0.0312, 0.86 ± 4.43 and 0.72 ± 0.0394 respectively. There were no significant differences between the OD of the cells in the groups that received doses of 1, 10, and 20 μM Res with the control group, but 50 μM of Res had a significant decrease (*P*< 0.05) (Figure 5).

### TUNEL staining

**Figure 6:**
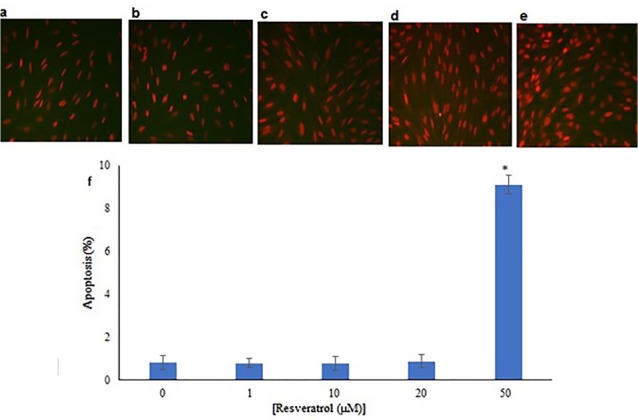
(a) Control group that were only incubated in the CDM. (b, c, d, e) CDM plus different doses of Res (1, 10, 20 and 50 μM) and (f) related diagram. Data were expressed as apoptotic cells percentage ± SD. Data were compared to the control group and *P*< 0.05.

TUNEL staining showed that the percentage of apoptotic cells in the control group and experimental groups (1, 10, 20 and 50 μM Res) were 0.83% ± 0.31, 0.80% ± 0.22, 0.78% ± 0.32, 0.88% ± 0.30 and 9.11% ± 0.44 respectively. There were no significant differences between the number of apoptotic cells in the groups of 1, 10 and 20 μM Res with the control group, but the group that received 50 μM of Res had a significant difference from the control group (*P*<0.05) (Figure 6).

### AO/EB double staining

**Figure 7:**
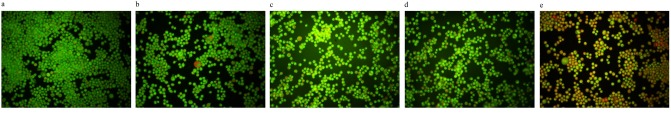
AO/EB staining, after 72 hours in a: control group that received only CDM, b: The group receiving 1 μM, c: 10 μM, d: 20 μM and e: 50 μM of Res plus the CDM respectively. Images were prepared using a fluorescence microscope, the healthy cells in green, the early stages of cell apoptosis in yellow and the late stages of cells apoptosis were showed in orange color.

The morphological changes of dead cells in AO/EB staining, in addition to fluorescent color changes, included cellular contractions, nucleuses fragmentation, and chromatin condensation, which were assessed. The number of dead cells was higher in the 50 μM Res dose, and most of them were in the early stages of apoptosis. There was a significant increase in comparison to the control group (*P*< 0.05) (Figure 7e), but there were no significant differences between the dead cells of 1, 10 and 20 μM Res doses with the control group (Figure 7a).

### FRAP

**Figure 8:**
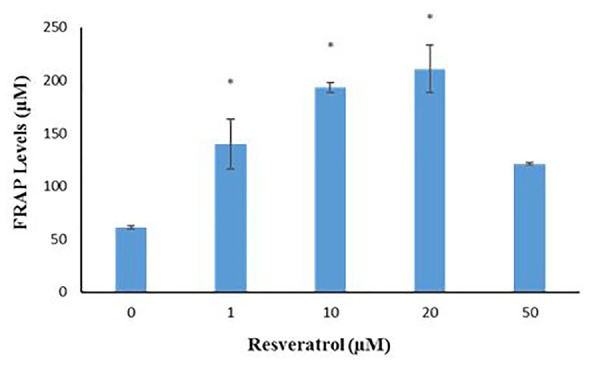
Levels of total antioxidant capacity in different groups with different doses of Res (0, 1, 10, 20, and 50 μM) were measured. Data were expressed as ± SD and compared to the control group, *P* = 0.00.

The FRAP levels (measurement of total antioxidant capacity) showed that in the control group and other groups with different doses of 1, 10, 20 and 50 μM Res were 60.9 ± 0.52, 139.45 ± 23.64, 192.95 ± 4.76, 210.95 ± 22.60 and 121.25 ± 1.30 respectively. There was a significant increase in FRAP amount in the groups of 1, 10 and 20 μM Res compared with the control group (*P*= 0), but there were no significant differences between the groups that received 50 μM of Res in comparison to the control group (*P*< 0.05) (Figure 8).

### Genes expression

**Figure 9:**
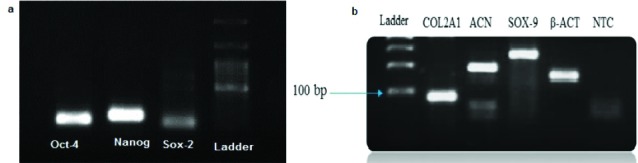
Gene’s expression (a) ASCs in the 4th passage and (b) chondrocyte derived from the differentiation of ASCs on day 21.

**Figure 10:**
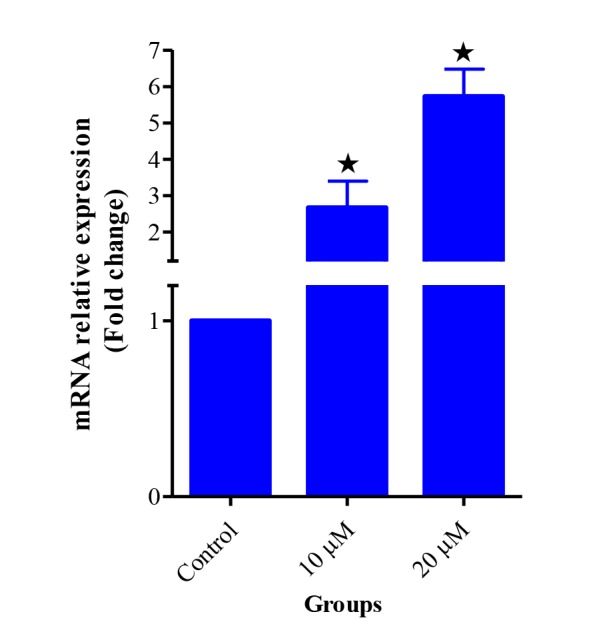
Gene expression of Sirt1 in the ASCs derived chondrocyte treated with the doses of 0 (Control), 10 and 20 μM Res on day 21. Data were expressed compare to the control group, *P* < 0.05.

Expression of a series of genes from ASCs and chondrocytes was evaluated using reverse transcription-PCR (RT-PCR). Expression of *Sox-2, Oct-4* and *Nanog* genes in ASCs, and *Sox-9,COL2A1* and *ACAN* genes in the chondrocyte were observed (Figure 9). The expression of Sirt1 gene was assessed using real-time PCR in 10 and 20 μM groups of Res and compared to the control group. The results showed that the expression of *Sirt1* gene in groups with doses of 10 and 20 μM Res had significantly increased (Figure 10).

## Discussion

In this study, the effect of Res on the differentiation of ASCs into chondrocyte in 3D culture was evaluated for the first time. ASCs were isolated from adipose tissue by collagenase digestion and cell filtrations through cell strainer. After confirming their stemness by FCM and expression some of related genes, the differentiation process occurred for 21 days. After differentiation, expression of cartilage specific genes (COL2A1, ACAN and Sox9) was confirmed. The effect of Res on ASCs differentiation to chondrocyte was dose-dependent, and 10 and 20 μM Res had the greatest effect on the number of these cells; however, a fatal effect at higher doses (50 μM) was seen.

Similar to the present study, other reports have been given on the efficacy of Res on MSCs.^32,33^ Its advantages include rapid efficacy in cell proliferation and differentiation, lack of toxicity and a simple and easy treatment.^34^ Peltz et al tested the effect of Res on the human MSCs in short and long time. Res increased self-renewal of cells by preventing their aging. Higher Res dose prevented the cell renewal by aging, stopping the cell cycle in phase S and increasing the doubling time of cells.^32^ We saw the same effect in high doses of Res. The difference between two studies was related to the kind of differentiation media and 2D culture vs. 3D culture condition that indicated better condition of cell maintenance.

Regarding the various biological properties of Res, there is still no report on its effect on ASCs being incubated in CDM. Res is an effective and well-informed compound that, if applied with a suitable dose and sufficient time, it can be used for cell therapy. The exact molecular mechanism of Res is not clear, but its possible pathways increase cell survival and longevity of the mammalian cells.^35^ Another possible mechanism for Res is protection of cell apoptosis.^36^ Referred to as physiological death, apoptosis contributes to the progress and homeostasis of the tissues and destroys the old and damaged cells.^37^ Any disturbances that occur in this process will lead to disease.^38^

Our results showed that the percentage of apoptotic cells in the 50 μM Res dose increased significantly compared to the control group, and most of them were in the early stage of apoptosis; but in the other doses of Res (1, 10 and 20 μM), however, the percentage decreased.

Research has shown that antioxidants as nutritional supplements increase the power of differentiation of these cells by preventing the destruction of cell DNA, reducing oxidative stress, and improving cell adhesion of culture media by enhancing the cell survival.^39^ Also, with a rise in the amount of antioxidants in the body, the diseases associated with ROS are reduced.^40,41^ Other studies have shown that natural compounds such as Res play an important role in reducing oxidative stress and related diseases.^42^ FRAP results of the present study indicated that the FRAP levels in doses of 1, 10 and 20 μM Res increased significantly compared to the control group.

TGF-β is one of the important factors in stem cells differentiation into chondrocyte, causing enhancement of the*COL2A1, ACAN* genes expression.^43^ This factor has a short half-life about three days and for more efficiency should be used freshly.^44^ Due to the increase in osteoarthritis in the world and the significant role that nutrition plays in the metabolism of chondrocytes, researchers use plant compounds for differentiation of stem cells into chondrocytes.

In recent years, different compounds such as soybeans and piascledine have been used for differentiation of MSCs to chondrocyte. The results of related studies have shown that both of them affected chondrogenesis and increased the expression of the *COL2A1, ACAN* genes, but this difference was not significant for soybeans compare to TGF-β, while there was a significant increase in the use of piascledine.^45,46^

As previously mentioned, Sirt1 has been implicated in damage to cellular DNA and carcinogens and protects cells against adverse conditions and cancers, therefore, increasing cell survival. In the present study, there was an upregulation of *Sirt1* gene expression in groups that received the appropriate dose of Res (10 and 20 µM). Gabay et al showed that the mice with Sirt1 defect were also defective in cartilage, and amount of their cartilage erosion progressed with increasing age. Therefore, normal cartilage homoeostasis requires the Sirt1 protein activity.^47^

Kim et al examined the effects of different doses of Res on normal and osteoarthritis human chondrocytes in 2D and 3D culture media incubated at 1, 10, 25 and 50 μM Res dose for 24, 48 and 72 hours. Their results showed that *Sirt1* gene was upregulated in the normal and osteoarthritis chondrocytes treated with 25 and 50 μM of Res at 72 hours.^48^ Therefore, the results of various studies indicated that the expression of the Sirt1 gene depends on the cell type, type of used substance and the duration of incubation

## Conclusion

3D culture was a suitable condition for ASCs differentiation to chondrocyte and lower doses of Res exert proliferation effect on ASCs. Res should be considered as a suitable differentiating compound in stem cell research and in cartilage repair.

## Ethical Issue

This study was approved by the Ethics Committee of Kermanshah University of Medical Sciences (No: 96396) and was done after obtaining the signed informed consent of the patients.

## Conflict of Interest

The authors declare that there is no conflict of interest in this study.

## Acknowledgments

This article is part of a PhD dissertation that was registered and sponsored by Kermanshah University of Medical Sciences (No. 96396).

## References

[1] Khazaei M, Bozorgi A, Khazaei S, Khademi A (2016). Stem cells in dentistry, sources, and applications. Dent Hypotheses.

[2] Friedenstein AJ, Chailakhjan RK, Lalykina KS (1970). The development of fibroblast colonies in monolayer cultures of guinea-pig bone marrow and spleen cells. Cell Tissue Kinet.

[3] Barry FP, Murphy JM (2004). Mesenchymal stem cells: clinical applications and biological characterization. Int J Biochem Cell Biol.

[4] Bourin P, Bunnell BA, Casteilla L, Dominici M, Katz AJ, March KL (2013). Stromal cells from the adipose tissue-derived stromal vascular fraction and culture expanded adipose tissue-derived stromal/stem cells: a joint statement of the International Federation for Adipose Therapeutics and Science (IFATS) and the International Society for Cellular Therapy (ISCT). Cytotherapy.

[5] Chan TM, Harn HJ, Lin HP, Chou PW, Chen JY, Ho TJ (2014). Improved human mesenchymal stem cell isolation. Cell Transplant.

[6] Nae S, Bordeianu I, Stăncioiu AT, Antohi N (2013). Human adipose-derived stem cells: definition, isolation, tissue-engineering applications. Rom J Morphol Embryol.

[7] Lin G, Garcia M, Ning H, Banie L, Guo YL, Lue TF (2008). Defining stem and progenitor cells within adipose tissue. Stem Cells Dev.

[8] Zuk PA, Zhu M, Ashjian P, De Ugarte DA, Huang JI, Mizuno H (2002). Human adipose tissue is a source of multipotent stem cells. Mol Biol Cell.

[9] Özen A, Gül Sancak İ (2014). Mezenkimal kök hücreler ve veteriner hekimLikte kullanımı. Ankara Üniv Vet Fak Derg.

[10] Sultan SM, Barr JS, Butala P, Davidson EH, Weinstein AL, Knobel D (2012). Fat grafting accelerates revascularisation and decreases fibrosis following thermal injury. J Plast Reconstr Aesthet Surg.

[11] Gelber AC, Hochberg MC, Mead LA, Wang NY, Wigley FM, Klag MJ (2000). Joint injury in young adults and risk for subsequent knee and hip osteoarthritis. Ann Intern Med.

[12] Ahmed TA, Hincke MT (2010). Strategies for articular cartilage lesion repair and functional restoration. Tissue Eng Part B Rev.

[13] Burns J, Yokota T, Ashihara H, Lean ME, Crozier A (2002). Plant foods and herbal sources of resveratrol. J Agric Food Chem.

[14] Sato M, Maulik G, Bagchi D, Das DK (2000). Myocardial protection by protykin, a novel extract of trans-resveratrol and emodin. Free Radic Res.

[15] Borrás C, Gambini J, Gómez-Cabrera MC, Sastre J, Pallardó FV, Mann GE (2006). Genistein, a soy isoflavone, up-regulates expression of antioxidant genes: involvement of estrogen receptors, ERK1/2, and NFkappaB. FASEB J.

[16] Yu W, Fu YC, Wang W (2012). Cellular and molecular effects of resveratrol in health and disease. J Cell Biochem.

[17] Liao PC, Ng LT, Lin LT, Richardson CD, Wang GH, Lin CC (2010). Resveratrol arrests cell cycle and induces apoptosis in human hepatocellular carcinoma Huh-7 cells. J Med Food.

[18] Dull AM, Moga MA, Dimienescu OG, Sechel G, Burtea V, Anastasiu CV (2019). Therapeutic approaches of resveratrol on endometriosis via anti-inflammatory and anti-Angiogenic pathways. Molecules.

[19] Frye RA (2000). Phylogenetic classification of prokaryotic and eukaryotic Sir2-like proteins. Biochem Biophys Res Commun.

[20] Matsuzaki T, Matsushita T, Takayama K, Matsumoto T, Nishida K, Kuroda R (2014). Disruption of Sirt1 in chondrocytes causes accelerated progression of osteoarthritis under mechanical stress and during ageing in mice. Ann Rheum Dis.

[21] Picard F, Kurtev M, Chung N, Topark-Ngarm A, Senawong T, Machado De Oliveira R (2004). Sirt1 promotes fat mobilization in white adipocytes by repressing PPAR-gamma. Nature.

[22] Firestein R, Blander G, Michan S, Oberdoerffer P, Ogino S, Campbell J (2008). The SIRT1 deacetylase suppresses intestinal tumorigenesis and colon cancer growth. PLoS One.

[23] Wang RH, Sengupta K, Li C, Kim HS, Cao L, Xiao C (2008). Impaired DNA damage response, genome instability, and tumorigenesis in SIRT1 mutant mice. Cancer Cell.

[24] Herranz D, Muñoz-Martin M, Cañamero M, Mulero F, Martinez-Pastor B, Fernandez-Capetillo O (2010). Sirt1 improves healthy ageing and protects from metabolic syndrome-associated cancer. Nat Commun.

[25] Zhao S, Xu W, Jiang W, Yu W, Lin Y, Zhang T (2010). Regulation of cellular metabolism by protein lysine acetylation. Science.

[26] Wang Q, Zhang Y, Yang C, Xiong H, Lin Y, Yao J (2010). Acetylation of metabolic enzymes coordinates carbon source utilization and metabolic flux. Science.

[27] Esfandiari N, Khazaei M, Ai J, Nazemian Z, Jolly A, Casper RF (2008). Angiogenesis following three-dimensional culture of isolated human endometrial stromal cells. Fertil Steril.

[28] Khazaei M, Pazhouhi M (2019). Induction of apoptosis and inhibition of autophagy cell death in the human prostate cancer cell lines by Trifolium pratens L hydroalcoholic extract. World J Cancer Res.

[29] Khazaei M, Pazhouhi M (2018). Protective effect of hydroalcoholic extracts of Trifolium pratense L on pancreatic beta cell line (RIN-5F) against cytotoxicty of streptozotocin. Res Pharm Sci.

[30] Khazaei M, Pazhouhi M (2019). Khazaei M, Pazhouhi MAntiproliferative effect of Trifolium pratens Lextract in human breast cancer cells. Nutr Cancer.

[31] Ghanbari E, Nejati V, Khazaei M (2016). Antioxidant and protective effects of Royal jelly on histopathological changes in testis of diabetic rats. Int J Reprod Biomed (Yazd).

[32] Peltz L, Gomez J, Marquez M, Alencastro F, Atashpanjeh N, Quang T (2012). Resveratrol exerts dosage and duration dependent effect on human mesenchymal stem cell development. PLoS One.

[33] Fischer-Posovszky P, Kukulus V, Tews D, Unterkircher T, Debatin KM, Fulda S (2010). Resveratrol regulates human adipocyte number and function in a Sirt1-dependent manner. Am J Clin Nutr.

[34] Signorelli P, Ghidoni R (2005). Resveratrol as an anticancer nutrient: molecular basis, open questions and promises. J Nutr Biochem.

[35] Yoon DS, Choi Y, Choi SM, Park KH, Lee JW (2015). Different effects of resveratrol on early and late passage mesenchymal stem cells through beta-catenin regulation. Biochem Biophys Res Commun.

[36] Jiang H, Zhang L, Kuo J, Kuo K, Gautam SC, Groc L (2005). Resveratrol-induced apoptotic death in human U251 glioma cells. Mol Cancer Ther.

[37] Israels ED, Israels LG (2000). The cell cycle. Oncologist.

[38] Ghavami S, Hashemi M, Ande SR, Yeganeh B, Xiao W, Eshraghi M (2009). Apoptosis and cancer: mutations within caspase genes. J Med Genet.

[39] Shaban S, El-Husseny MWA, Abushouk AI, Salem AMA, Mamdouh M, Abdel-Daim MM (2017). Effects of antioxidant supplements on the survival and differentiation of stem cells. Oxid Med Cell Longev.

[40] Sen S, Chakraborty R (2011). The role of antioxidants in human health. ACS Symposium Series.

[41] Lobo V, Patil A, Phatak A, Chandra N (2010). Free radicals, antioxidants and functional foods: Impact on human health. Pharmacogn Rev.

[42] Jiménez-Estrada M, Velázquez-Contreras C, Garibay-Escobar A, Sierras-Canchola D, Lapizco-Vázquez R, Ortiz-Sandoval C (2013). In vitro antioxidant and antiproliferative activities of plants of the ethnopharmacopeia from northwest of Mexico. BMC Complement Altern Med.

[43] Thorpe SD, Buckley CT, Vinardell T, O’Brien FJ, Campbell VA, Kelly DJ (2010). The response of bone marrow-derived mesenchymal stem cells to dynamic compression following TGF-beta3 induced chondrogenic differentiation. Ann Biomed Eng.

[44] Saraf A, Mikos AG (2006). Gene delivery strategies for cartilage tissue engineering. Adv Drug Deliv Rev.

[45] Bamdadpasand Shekarsaraei F, Eslami Farsani M, Heydarieh N (2017). The Effect of Soy Isoflavone on the Proliferation and Differentiation of Adipose-Derived Mesenchymal Stem Cells into Chondrocytes and Expression of Collagen II and Aggrecan Genes. Qom Univ Med Sci J.

[46] Esmaeily M, Hashemibeni B, Valiani A, Amirpour N, Purmollaabbasi B, Kazemi M (2016). Effect of Piasclidin on Induction of Chondrogenesis by Human Adipose-Derived Stem Cells in Fibrin Scaffold. Journal of Isfahan Medical School.

[47] Gabay O, Sanchez C, Dvir-Ginzberg M, Gagarina V, Zaal KJ, Song Y (2013). Sirtuin 1 enzymatic activity is required for cartilage homeostasis in vivo in a mouse model. Arthritis Rheum.

[48] Kim HJ, Braun HJ, Dragoo JL (2014). The effect of resveratrol on normal and osteoarthritic chondrocyte metabolism. Bone Joint Res.

